# Evaluation of Physicochemical Properties and Prebiotics Function of a Bioactive *Pleurotus eryngii* Aqueous Extract Powder Obtained by Spray Drying

**DOI:** 10.3390/nu16111555

**Published:** 2024-05-21

**Authors:** Jianqiu Chen, Mengling Zhou, Liding Chen, Chengfeng Yang, Yating Deng, Jiahuan Li, Shujing Sun

**Affiliations:** 1 College of Life Sciences, Fujian Agriculture and Forestry University, Fuzhou 350002, China; cjqsw05@fafu.edu.cn (J.C.); 3210537056@fafu.edu.cn (M.Z.); chenliding@fafu.edu.cn (L.C.); 12305014041@fafu.edu.cn (Y.D.); 2 Gutian Edible Fungi Research Institute, Fujian Agriculture and Forestry University, Ningde 352200, China; 3 Sanya Institute, China Agricultural University, Sanya 572025, China; cfyang07@cau.edu.cn

**Keywords:** *Pleurotus eryngii* aqueous extract, physicochemical property, antioxidant activity, modulating intestinal microbiota

## Abstract

A bioactive *Pleurotus eryngii* aqueous extract powder (SPAE) was obtained by spray drying and its performance in terms of physicochemical properties, in vitro digestion, inflammatory factors, and modulation of the intestinal microbiota was explored. The results indicated that the SPAE exhibited a more uniform particle size distribution than *P. eryngii* polysaccharide (PEP). Meanwhile, a typical absorption peak observed at 843 cm^−1^ in the SPAE FTIR spectra indicated the existence of α-glycosidic bonds. SPAE exhibited higher antioxidant abilities and superior resistance to digestion in vitro. In addition, SPAE supplementation to mice significantly reduced the release of factors that promote inflammation, enhanced the secretion of anti-inflammatory factors, and sustained maximum production of short-chain fatty acids (SCFAs). Additionally, it significantly enhanced the relative abundance of SCFAs-producing *Akkermansia* and reduced the abundance of *Ruminococcus* and *Clostridiides* in intestines of mice. These results show the potential of SPAE as a novel material with prebiotic effects for the food and pharmaceutical industries.

## 1. Introduction

*P. eryngii* is one of the most extensively cultivated edible fungi in the global market [1]. Polysaccharides are the primary component of *P. eryngii*, which have the advantage of high efficacy in low dose and convenient edibility compared to *P. eryngii* itself, and they have a variety of bioactivities, including antioxidant, antihyperglycemic, antitumor, antiaging, immunomodulatory, bacteriostatic, and bifidogenic effects [2,3]. Accordingly, polysaccharides derived from the mycelium and fruiting body of *P. eryngii* have promising applications in both functional foods and medicines [3,4,5]. At present, the conventional method for preparing PEP involved ethanol precipitation and freeze drying [5], resulting in a complex process, high costs, strong hygroscopicity, storage challenges, and limitations in food applications due to the use of ethanol [6]. SPAE is an extract obtained by spray drying with polysaccharides as the main component. Spray drying has the advantages of short drying time, simple process, and controllability. It is also a fast and cost-effective drying strategy with high production capacity, making it a promising alternative to the freeze drying method commonly used in industrial production [7,8,9]. To our knowledge, there are limited reports on how drying methods (especially spray drying) affect physicochemical properties of active substance in *P. eryngii* and their in vivo bioactivities [10,11].

Gut microbiota is critical in maintaining digestive function, immune system regulation and pathogen resistance [12]. An imbalance in the gut microbiota can break the relationship between gut microbes and their hosts, leading to a range of diseases. Thus, a dynamic balance of the gut microbiota is crucial for human health [13]. The market demand for prebiotics is increasing due to their essential role in maintaining a healthy balance of intestinal microorganisms and enhancing general health [14]. Previous research has indicated that polysaccharides may have prebiotic activity, which could enhance the growth of beneficial bacterial microbiota, such as *Lactobacillus* and *Bifidobacterium*, thereby improving intestinal health. They additionally promote the synthesis of advantageous metabolites, including SCFAs, by gut microbes [15,16].

Gut microbes have been reported to be able to generate SCFAs and regulate intestinal microbiota composition by utilizing PEP during in vitro digestion [5,17]. This demonstrated that PEP had prebiotic potentials. Therefore, the objective of this study was to develop a material with similar biological activity to PEP, but with lower cost, stable and powdered. Consequently, SPAE was produced through spray drying, and its physicochemical properties and probiotic function were subsequently determined. The structural properties of SPAE were investigated, including analysis of scanning electron microscopy (SEM), Fourier transform infrared spectroscopy (FT-IR), thermogravimetric analysis (TGA), and differential scanning calorimetry (DSC). A dynamic in vitro digestion model was used to illustrate the changes in the in vitro digestion behavior of SPAE. In addition, the prebiotic potency of SPAE was assessed by investigating their effects on the mice intestinal microbiota, and the key microbiota was revealed by analyzing the correlation of microbiota and biochemical indexes (serum immunity indexes and SCFAs). The results of this work will reveal a bioactive aqueous extract from *P. eryngii* with prebiotic functionality and extend the potential applications to functional food and pharmaceutical sectors.

## 2. Materials and Methods

### 2.1. Materials

Dried *P. eryngii* fruiting bodies were purchased from Gutian County, Fujian Province, China. 2,2-Diphenyl-1-picrylhydrazyl (DPPH), 3,5-Dinitrosalicylic acid (DNS), α-amylase, pepsin, mucin, pancreatin, bile salts, and volatile free acid mixture (SCFA) standards were purchased from Sigma-Aldrich Co. Ltd. (St. Louis, MO, USA). 2,2′-Azinobis [3-ethylbenzothiazoline-6-sulfonic acid]-diammonium salt (ABTS) was purchased from Macklin, Inc. (Shanghai, China). Ethanol was purchased from the Aladdin Bio-Chem Technology Co. Ltd. (Shanghai, China). All other chemicals were of analytical grade and purchased from Sinopharm Co. Ltd. (Shanghai, China).

### 2.2. Preparation of SPAE and PEP

Dried *P. eryngii* fruit bodies (500 g) were washed and placed into a 60-mesh filter bag in a 50 L decompressing extraction and concentrating unit (Weifang Beifang Pharmaceutical Equipment Manufacturing Co., Ltd., Weifang, Shandong Province, China) to extract and concentrate. The ratio of material to liquid was 1:60 (*w*/*v*) and the extraction temperature was 80–90 °C. Two extractions were carried out under negative pressure for 3 h each, the extracts were mixed and then concentrated under negative pressure to 1/4 of the original extract, and finally the concentrated liquid was used for spray drying. The inlet air temperature of the LPG-5 centrifugal spray dryer (Weifang Beifang Pharmaceutical Equipment Manufacturing Co., Ltd., Weifang, Shandong Province, China) was set to 180 °C, the outlet temperature was 90 °C, the peristaltic pump was set to 20 rpm, and SPAE was obtained after drying the concentrate. PEP was prepared according to previously reported methods [5].

### 2.3. Characterization of SPAE and PEP 

#### 2.3.1. SEM

SPAE and PEP samples were ground and sifted through 100 mesh to perform SEM observation (Thermo Scientific Phenom ProX, Waltham, MA, USA) according to Chen et al. [18]. Briefly, the samples were gold plated and then examined in the SEM at an acceleration voltage of 15 kV, with magnifications of 10,000×, 5000×, 2500×, and 1000×.

#### 2.3.2. FTIR

SPAE and PEP samples were analyzed using the spectrophotometer (Thermo Fisher Nicolet iS10, Waltham, MA, USA) with the wavelength range recorded spanning 4000 to 400 cm^−1^ [19,20].

#### 2.3.3. TGA and DSC

Thermogravimetric analysis of SPAE and PEP was performed using the thermal analyzer (Netzsch TG209F3, Netzsch Corporation, Bavaria, Germany) according to previously reported methods [20]. Briefly, the two samples were subjected to gradual heating in a nitrogen atmosphere, ranging from 50 to 650 °C with the rate 5 °C per minute, and TGA–DTG and DSC curves were obtained.

### 2.4. Determination of Antioxidant Activity of SPAE and PEP In Vitro

The ABTS radical and DPPH radical scavenging activities of SPAE and PEP were determined according to previously reported methods [20], and, finally, the concentration of the samples required to achieve a scavenging rate of 50% was calculated, namely, EC_50_ value.

### 2.5. In Vitro Digestion of SPAE and PEP

Simulated saliva fluid (SSF) and simulated gastric fluid (SGF) for dynamic in vitro digestion were prepared according to previously described methods [20,21]. SGF pH was set to 1.6 by using 1 M HCl. Simulated intestinal fluid (SIF) was prepared by dissolving pancreatin (9.46 mg per gram of dry sample), bile salt (28.38 mg per gram of dry sample), and NaHCO_3_ (4.5 mg/mL) in distilled water with adjusted pH of 7.50 using 1 M NaOH [22]. SPAE and PEP solutions were of 32 mg/mL. The enzymes in all samples were heat-treated at 100 °C in 15 min for inactivation, then reducing sugar content in the samples were determined [20,23].

### 2.6. Animal Experiments

#### 2.6.1. Experimental Design for Animals

Six-week-old male ICR mice weighing 18–22 g were supplied by SPF Biotechnology Co. Ltd. (Beijing, China; SCXK (Jing) 2016-0002). The diets were purchased from HFK Bioscience Co., Ltd. software (Beijing, China). All mice were given 10 days to acclimate to new housing conditions of 25 ± 2 °C and following a 12/12 h light–dark cycle. They had unrestricted access to food and water before the experiment began. 

Thirty mice were distributed randomly into five treatment categories (n = 6), and maintained for 5 weeks on control treatment (Con), low dose of SPAE treatment (SPAE-L, 100 mg/kg body weight), high dose of SPAE treatment (SPAE-H, 400 mg/kg body weight), low dose of PEP treatment (PEP-L, 100 mg/kg body weight) and high dose of PEP treatment (PEP-H, 400 mg/kg body weight), and the mice were gavaged with equal amounts of SAPE solution, PEP solution, or distilled water. At the end of the 5-week experiment, all mice were killed for cervical dislocation after carbon dioxide inhalation anesthesia. The serum, various organs (brain, liver, spleen, thymus, pancreas), and the contents of the mice cecum were collected and frozen using liquid nitrogen, and then preserved at −80 °C [18,24]. 

#### 2.6.2. Determination of Organ Indexes in Mice

Body weights were measured daily to monitor mice growth. The weights of various organs (brain, liver, spleen, thymus, and pancreas) in each mice were measured at the end, and the corresponding organ indices were calculated [25].

#### 2.6.3. Determination of Biochemical Indices in Mice

The cecum contents from mice were dissolved using distilled water with 1:10 ratio (*w*/*v*) and mixed in 10 mL centrifuge tubes to ensure uniformity. Then, the samples were kept for a 20 min ice bath prior to measurement, and a pH meter (PHS-3E pH-meter, INASE Scientific Instrument Co., Ltd., Shanghai, China) was used to determine the pH of the mice intestines.

The ELISA kits (Jiangsu Meimian Industrial Co., Ltd, Yancheng, Jiangsu Province, China) were employed to assess levels of proinflammatory cytokines (IL-1, IL-6, and TNF-α), and anti-inflammatory cytokine (IL-10).

#### 2.6.4. Determination of SCFAs in Mice Intestines

The sample solution was made with 50 mg of cecum contents dissolved in 500 µL of water and sonicated for 10 min. After adding 500 μL methanol for vortexing, the solution was centrifuged for 5 min with 12,000 rpm at 4 °C. Then, 50 µL 3-nitrophenylhydrazine (250 mM) and 50 µL 1-(3-dimethylaminopropyl)-3-ethylcarbodiimide hydrochloride (150 mM) was added to 50 µL supernatant, mixed, and derivatized under 30 °C for 30 min. Subsequently, 50 μL of 2,6-di-tert-butyl-para-cresol (BHT) methanol solution (2 mg/mL) and 250 μL of 75% methanol solution were combined and spun at 12,000 rpm for 5 min under 4 °C. 

SCFAs were analyzed using ultra-high-performance liquid chromatography–mass spectrometry (UPLC-MS) (Waters, Milford, MA, USA). The column model was Waters UPLC BEH C8. The column temperature was maintained at 45 °C, with an injection volume of 5 µL and a flow rate of 0.3 mL/min. The composition of the mobile phase included 0.01% formic acid along with a blend of methanol and isopropyl alcohol in 8:2 *v*/*v* ratio. The voltage and temperature were 3.0 kV and 150 °C. The desolvation process took place at 450 °C, with a gas flow rate of 1000 L/h and 10 L/h for the cone gas flow rate. TargetLynx tool of MassLynx 4.2 Software was used to calculate the target data peak areas with a retention time error of 15 s. Quantitative results were obtained using a standard curve method.

#### 2.6.5. Cecal Contents DNA Extraction and 16S High-Throughput Sequencing

Genomic DNA of the cecal contents was extracted by the E.Z.N.A™ Mag-Bind Soil DNA Kit (Omega bio-tek, Shanghai, China) DNA integrity was detected by agarose gel, and the concentration of DNA samples was quantified by ThermoFisher Qubit^®^ 4.0 fluorometer (MA, USA). PCR was used to amplify the V3-V4 (341F: CCTACGGGNGGCWGCAG, 805R: GACTACHVGGTATCTAATCC) hypervariable region of the 16S rRNA gene, and the DNA of the target region was amplified and enriched in order to be used for high-throughput sequencing by using the Illumina Miseq sequencing platform (Illumina, Inc., San Diego, CA, USA). The sequencing data were provided by Sangon Biotech Co., Ltd. (Shanghai, China). The data were analyzed with reference to previous studies [5,26,27]. A 97% similarity clustering of sequences was performed in operational taxonomic units (OTUs) clustering after extraction of nonrepetitive sequences for subsequent species annotation. The database used was the Ribosomal Database Project (RDP database).

### 2.7. Statistical Analysis

All tests were conducted in at least triplicate and the results presented as mean ± standard deviation. Statistical analysis was performed using one-way analysis of variance (ANOVA). SPSS Statistics 22 and Origin 8.0 software were used for plotting. Analysis of microorganisms was carried out using statistical software R 4.2.2.

## 3. Results and Discussion

### 3.1. Characterization of SPAE

#### 3.1.1. Morphological Properties

**Figure 1:**
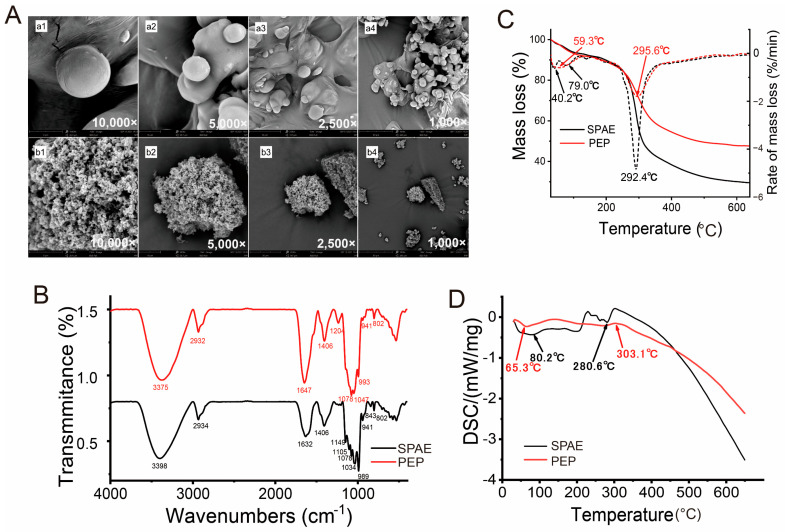
Characterization of the SPAE and PEP. (**A**) Scanning electron micrographs (**a1**–**a4**: SPAE, **b1**–**b4**: PEP). (**B**) FTIR spectra. (**C**) TGA–DTG curves. (**D**) DSC curves.

As depicted in Figure 1A, SPAE exhibited a more uniform particle size distribution and a more spherical shape than the PEP, aligning with earlier research [17]. The smoother surface of SPAE was attributed to the turbulence during the spray drying process, leading to some erosion of the particles [9]. The particle morphology of PEP was irregular, porous, and loose due to the prefrozen ice crystals of PEP sublimating directly into water vapor during the freeze drying process. Moreover, it was observed that the SPAE particles were connected and formed a tight, uniform, spherical chain conformation with an island-shaped structure, while no bonds were formed between the PEP particles. This could be a result of the molecular aggregation caused by the hot processing of spray drying [28].

#### 3.1.2. FT-IR Spectra Analysis

The FT-IR spectra of SPAE and PEP are depicted in Figure 1B. The absorption peaks at 3375 and 3398 cm^−1^ corresponded to O-H stretching vibrations, suggesting the presence of hydroxyl groups in both SPAE and PEP samples. The two absorption peaks at 2932 and 2934 cm^−1^ were attributed to C-H stretching vibrations. Moreover, it was commonly believed that the bands represented the typical peaks of polysaccharides [28], indicating that SPAE retained the characteristic peaks of polysaccharides as PEP did. The carbonyl asymmetric stretching vibration absorption peaks at 1647 and 1632 cm^−1^ indicated the presence of uronic acid in SPAE and PEP. Although the skeletal structure of SPAE was similar to PEP, some characteristic peaks were observed between the two samples. For example, PEP had the absorption peaks at 1204 cm^−1^ representing the C-O stretching band associated with the C-O-SO_3_ group, while the absorption peaks at 1149 and 1105 cm^−1^ were typical signals of the pyranose ring of SPAE. A blue shift was observed in SPAE at 1047 and 993 cm^−1^ in comparison to the PEP, resulting in the absorption peaks at 1034 and 959 cm^−1^ of SPAE, which might be due to thermal aggregations of PEPs [28]. Furthermore, it was observed that only SPAE exhibited absorption peaks at 843 cm^−1^, indicating the presence of a small amount of α-glycosidic bond.

#### 3.1.3. Thermogravimetric Analysis

The TGA–DTG curves of SPAE and PEP are displayed in Figure 1C. The heat loss trends of the two samples were consistent. Water loss was caused by heating at 40.2 °C, 79.0 °C, and 59.3 °C. SPAE and PEP experienced the second mass loss with maximum weight loss temperatures of 292.4 °C and 295.6 °C, respectively. As a result, there was 70.64% and 52.43% mass loss, which could be attributed to depolymerization of the polysaccharide structure. Additionally, SPAE exhibited shorter molecular chains and lower molecular weight after drying, making it more prone to thermal decomposition and resulting in greater mass loss within a narrow temperature range. In contrast, the PEP had longer molecular chains and a higher molecular weight after lyophilization, which might prevent the molecular chains from breaking during heating [7,29,30]. As shown in Figure 1D, the endothermic peaks at 65.3 °C and 80.2 °C were caused by the loss of water during the heating process. A small exothermic peak was observed for PEP at 303.1 °C, indicating its decomposition during heating. However, the SPAE curve showed a small endothermic peak at 280.6 °C, suggesting that SPAE may undergo a transition from a solid to an amorphous liquid during decomposition [31]. 

### 3.2. Antioxidative Activity In Vitro

**Figure 2:**
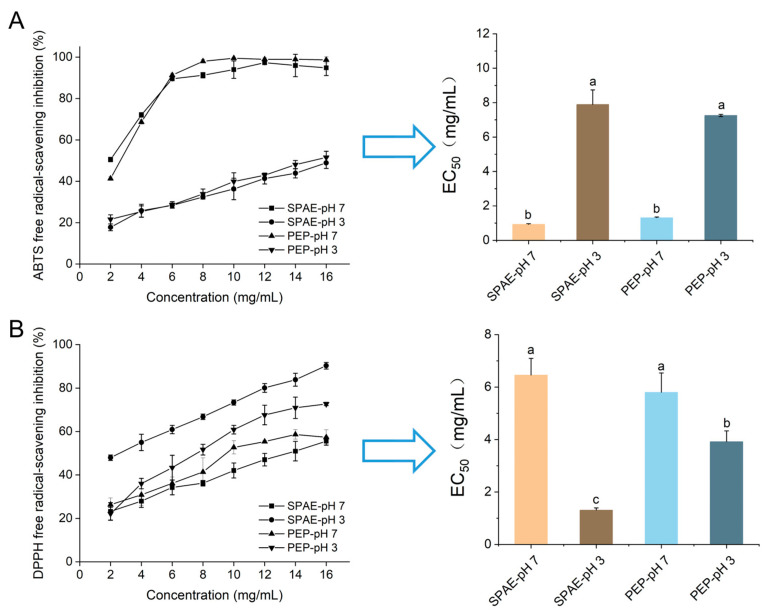
ABTS (**A**) and DPPH (**B**) free radical scavenging effects of SPAE and PEP at different pH. The use of lowercase letters indicates significant differences among the treatment groups at a significance level of *p* < 0.05.

The impact of pH on the antioxidative properties of SPAE and PEP was displayed in Figure 2. The two samples showed similar scavenging effects at different pH values, which could be related to the similar characteristic functional groups according to the above study. The ABTS free radical quenching rate of two samples was higher at pH 7, whereas they had a better quenching effect on DPPH free radicals at pH 3. Notably, SPAE exhibited superior DPPH antioxidant activity and a lower EC_50_ value (1.30 mg/mL) at pH 3 (Figure 2B) compared to PEP. This finding was consistent with a previous study that showed that some heterocyclic compounds, including furans, thiophene, and pyrazines, could improve the antioxidant activity of *Perinereis aibuhitensis* hydrolysates after spray drying [29]. Therefore, it indicated that the antioxidation activity of SPAE held great potential in the food industry, particularly for acid foods, which was attributed to the lower EC_50_ value of DPPH under acidic conditions (pH 3).

### 3.3. Variations in Reducing Sugar of SPAE during Dynamic Digestion In Vitro

The variations in reducing sugar content during dynamic in vitro digestion of SPAE are presented in Table S1. Generally, the breakdown of the polymer and glycosidic linkages in polysaccharides was associated with the gastric acidic environment, leading to a higher content of reducing sugar [32]. The reducing sugar content increased slightly during dynamic in vitro digestion. The concentration of reducing sugar in SPAE was lower than PEP during the gastric phase, indicating that SPAE had better resistance to the upper digestive tract. This suggested that SPAE retained more active polysaccharides to reach the large intestine which may be utilized by gut microbes to generate prebiotic metabolites. Wu et al. [33] reported that okra pectin polysaccharide (OPP-D) was partially degraded during in vitro digestion and remarkably degraded and utilized by human intestinal microbiota with the promotion of SCFAs.

### 3.4. Effects of SPAE on the Gross Growth and Organ Indexes in Mice 

No abnormal behavior, treatment-related illness, or death occurred in any of the mice groups throughout the study. The mice grew normally, without diarrhea or constipation. The gross growth and organ indexes of the mice are shown in Figure S1, and the mice in all treatment groups showed no significant increase in body weight. The thymus index of SPAE-L and the spleen index of SPAE-H of mice treated were higher than those of the Con group. However, there were no changes in the indices of the brain, liver, and pancreas. The thymus and spleen are vital immune organs in the human body. Their indexes can reflect the toxicity of ingested substances and the state of the immune function to a moderate extent [25]. The results indicated that within the experimental concentration range, SPAE exhibited no toxic effects and might have the potential to enhance the immune activity of mice.

### 3.5. Effects of SPAE on Inflammatory Parameters in Serum of Mice

**Figure 3:**
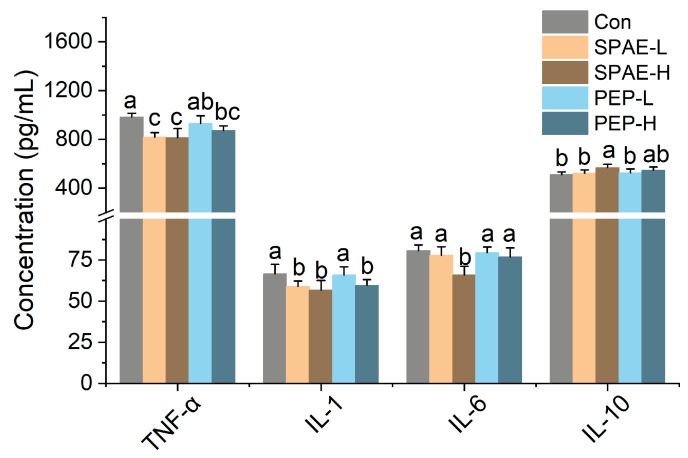
Effects of SPAE and PEP on inflammatory parameters in serum of mice. The use of lowercase letters indicates significant differences among the treatment groups at a significance level of *p* < 0.05.

Inflammation has been considered to be an automatic defense response of the body’s immune system against adverse stimuli [34]. The extracts of fungi, as a natural immune modulator, could effectively alleviate the occurrence of inflammation through modulating the secretion of inflammatory factors [35]. As presented in Figure 3, a decrease in the expression of interleukin-1 (IL-1), interleukin-6 (IL-6), and tumor necrosis factor alpha (TNF-α) were observed in the groups treated with two samples compared with the Con group, and anti-inflammatory factor interleukin-10 (IL-10) of SPAE-H was markedly increased. The post-intervention effect was more pronounced in the SPAE-H group (*p* < 0.05), suggesting that SPAE might have a potentially more powerful immunomodulatory effect. 

### 3.6. Effects of SPAE on Intestinal pH and SCFAs Concentration

**Figure 4:**
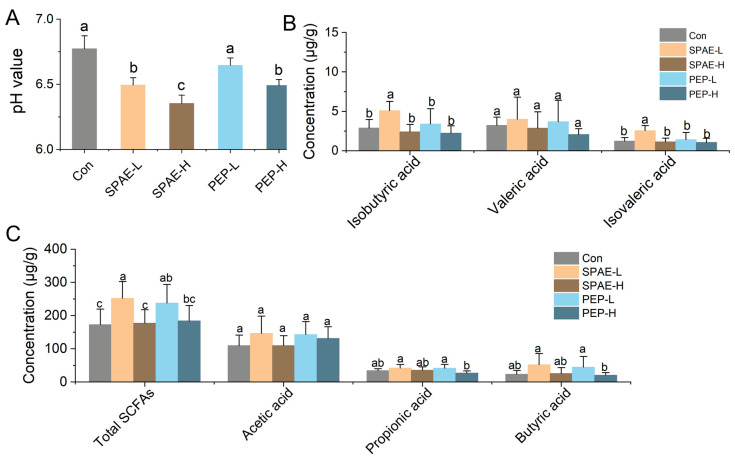
Concentrations of SCFAs in intestinal tract of mice with SPAE and PEP. (**A**) pH value. (**B**) and (**C**) intestinal SCFAs. The use of lowercase letters indicates significant differences among the treatment groups at a significance level of *p* < 0.05.

SCFAs are produced by the intestinal microbiota through the degradation of polysaccharides, resulting in pH reduction and affecting intestinal microbiota composition [5]. SCFAs are crucial for the host’s metabolism and immune system [36]. As shown in Figure 4A, the intestinal pH of SPAE-H was the lowest at 6.35, followed by SPAE-L (6.49), which were significantly lower than Con group (*p* < 0.05). SPAE groups always had a lower pH value than that of PEP groups under the same conditions. The results indicated that SPAE supplementation produced SCFAs and other products, leading to a lower pH. Previous studies have demonstrated that a weakly acidic condition could stimulate beneficial bacteria growth while inhibiting harmful bacteria proliferation [37]. 

The levels of SCFAs in the intestine of mice are illustrated in Figure 4B,C. Compared to the PEP groups, the SPAE groups showed an equal ability to produce SCFAs. The total concentration of SCFAs increased in SPAE-L and PEP-L more than the Con group (*p* < 0.05). It was observed that the concentrations of isobutyric acid and isovaleric acid in SPAE-L groups were the highest and significantly increased more than other groups (*p* < 0.05). Increased levels of butyric acid and isobutyric acid alleviated DSS-induced ulcerative colitis [38]. Isovaleric acid alleviates ovariectomy-induced osteoporosis by inhibiting osteoclast differentiation [39]. It has been reported that the production of SCFAs was strongly correlated with structural features of materials [36,40]. The superior ability of SPAE to generate SCFAs may be attributed to the higher specific surface area of its particles, which were formed through spray drying [41]. It is worth noting that both SPAE and PEP did not show a dose-dependent manner in the production of SCFAs, and high concentrations of samples might inhibit the production of SCFAs. The results of pH reduction and SCFAs production were consistent with those reported by Ma et al. [5] for the characteristics during simulated gastrointestinal digestion and fermentation of *P. eryngii* polysaccharide (PEP).

### 3.7. Effects of SPAE on Intestinal Microbiota Composition Analysis

**Figure 5:**
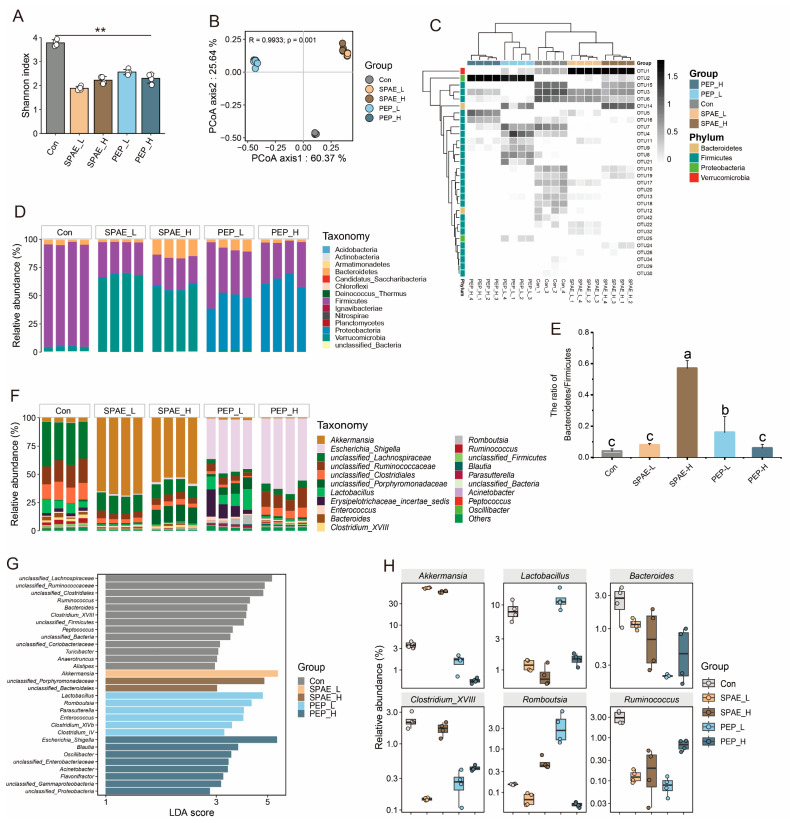
Effects of SPAE and PEP on gut microbiota composition analysis. (**A**) α diversity analyzed by Shannon index. (**B**) β diversity analyzed by PCoA. (**C**) OTU level of gut microbiota. (**D**) Phylum level of gut microbiota. (**E**) The ratio of *Bacteroidetes* and *Firmicutes*. (**F**–**H**) Genus level of gut microbiota. The use of “**” indicates significant differences among the treatment groups at a significance level of *p* < 0.01. The use of lowercase letters indicates significant differences among the treatment groups at a significance level of *p* < 0.05.

A total of 443 operational taxonomic units (OTUs) were detected by statistical and comparative analyses, out of which 325 OTUs were shared by all groups. As shown in Figure 5A, the α diversity analysis was performed according to Shannon index. The results of this study revealed a decrease in the enrichment and variety of gut bacteria in SPAE and PEP groups, in agreement with the previous study [42]. In this study, β diversity was evaluated by using principal coordinate analysis (PCoA). As demonstrated in Figure 5B, the horizontal (Axis 1, 60.37%) and vertical (Axis 2, 25.64%) axes were the two main components explaining the variance between samples, accounting for 86.01% of the total variance. Notably, the data points of the figure in the close proximity reflect smaller differences between samples. The distance between experimental groups with different doses was relatively close, indicating that the correlation between microbial diversity and dose was not significant, while the distance between SPAE groups, PEP groups, and Con group was longer, indicating that the microbiota compositions were altered after treatment with both SPAE and PEP.

The changes in OTU level of microbial communities after SPAE and PEP treatments were investigated, and a heatmap was employed to display correlations between key OTUs (Figure 5C). SPAE and PEP groups significantly regulated OTU levels of cecum microbiota in comparison with the Con group, including *Bacteroidetes*, *Firmicutes*, *Proteobacteria*, and *Verrucomicrobia*. The community structure of microorganisms was discussed at the phylum level and the analysis is presented in Figure 5D. The gut microbiota of mice was composed of five major phyla: *Firmicutes*, *Verrucomicrobia*, *Proteobacteria*, *Bacteroidetes*, and *Actinobacteria*. Compared with the Con group, the abundance of *Firmicutes* was decreased in all experimental groups, while the abundance of *Verrucomicrobia* increased most in the SPAE treatment groups, and the abundance of *Proteobacteria* increased the most in the PEP treatment groups. Consequently, the *Bacteroides* to *Firmicutes* ratio increased considerably (*p* < 0.05) (Figure 5E). The ratio in the SPAE-H group was the highest (0.57), followed by the PEP-L group (0.16). Previous studies have shown that *Bacteroidetes* could encode more carbohydrate-degrading enzymes than *Firmicutes*, which was beneficial for hydrolyzing digestible polysaccharides and promoting SCFAs production [43].

The top 20 genera for analysis are depicted in Figure 5F, indicating the differential enrichment of intestinal microbes at the genus level among mice in the various treatment groups. According to Figure 5G,H, the relative abundance of *Ruminococcus*, *Clostridium_XVIII*, *Peptococcus*, *Alistipes*, *Acinetobacter*, and *Anaerotruncus* in the four experimental groups all decreased in comparation with the Con group. However, the abundance of *Akkermansia* in two SPAE groups and *Lactobacillus* and *Romboutsia* in PEP-L group significantly increased (*p* < 0.05). 

*Akkermansia* belongs to the phylum *Verrucomicrobia*, with only one member called *Akkermansia muciniphila* [44]. Numerous studies have shown that increasing the abundance of *A. muciniphila* can reduce the incidence of diabetes, cardiovascular disease, intestinal disease, and neurological disorders. The evidence from animal and human studies indicated that *A. muciniphila* would be the next generation of probiotics with clinical application prospects, especially particularly in the prevention and treatment of diabetes, obesity, and cancer [45,46]. *Lactobacillus* is a probiotic that ferments carbohydrates to produce lactic acid, improving the intestinal mucosal barrier and promoting normal intestinal function [47]. Previous studies have indicated that adding *Lactobacillus* to the diet can reduce liver and intestinal inflammation in mice caused by *Escherichia coli* [48]. *Romboutsia* is commonly found in healthy human mucosa and may be associated with host health [49]. Meanwhile, it has been well documented that *Akkermansia*, *Lactobacillus*, and *Romboutsia* can degrade polysaccharides to produce SCFAs, maintaining host health [47,50]. Moreover, the relative abundance of *Clostridium_XVIII* and *Ruminococcus* was decreased significantly by SPAE and PEP. Previous study indicated that high concentrations of theasinensin could significantly reduce the relative abundance of *Clostridium_XVIII, Acetatifactor, and Anaerotruncus* that lead to intestinal dysbiosis, alleviating symptoms associated with diabetes [51], and high levels of *Ruminococcus* were also reported in the feces of HFD-fed mice [50], so the high abundance of *Clostridium_XVIII* and *Ruminococcus* is undesirable for human health, whereas *Escherichia-Shigella* was also a genus of higher abundance in the PEP treatment. *Escherichia-Shigella* is an opportunistic pathogen. PEP supplementation not only stimulated the growth of probiotics, but might also selectively promote the growth of some pathogenic bacteria. The above research suggests that SPAE can protect the intestines by increasing beneficial microbiota such as *Akkermansia* and reducing harmful bacteria such as *Clostridium_XVIII* and *Ruminococcus*, which is similar to the probiotic effect of PEP. This indicates that SPAE may have potential as a probiotic in promoting gut health. 

### 3.8. Relationship between Inflammatory Parameters, SCFAs and Microbiota

**Figure 6:**
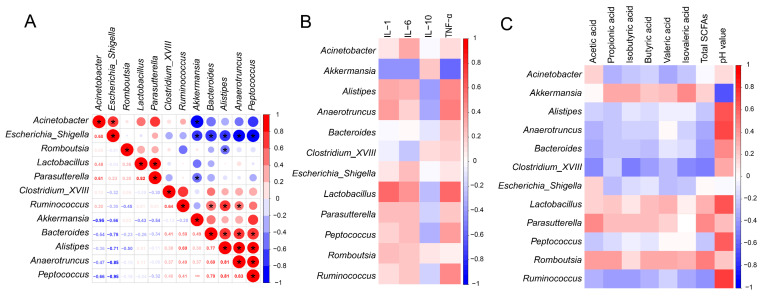
Relationship between inflammatory parameters, SCFAs, and gut microbiota. (**A**) Correlation analysis of gut microbiota at genus level. (**B**) Heatmap of the correlation coefficients between inflammatory parameters and gut microbiota at genus level. (**C**) Heatmap of correlation coefficients between SCFAs and gut microbiota at genus levels. The use of “*” indicates significant differences among the treatment groups at a significance level of *p* < 0.05.

The Spearman analysis method was adopted to analyze the genus-level relationships of microbial communities (Figure 6A). As the dominant genera in the SPAE groups, *Akkermansia* showed a significant negative correlation with harmful bacteria in *Acinetobacter* and *Escherichia-Shigella*, with correlation coefficients of −0.95 and −0.66, respectively. *Lactobacillus*, as the main genus in PEP group, showed a significant positive correlation with *Parasutterella*. *Parasutterella* have been reported to stably colonize the mice intestines and might also be concerned with the maintenance of bile acid homeostasis and metabolism of cholesterol [52].

The correlation between the gut microbiota (Top 20), Inflammatory parameters, and SCFAs is shown in Figure 6B. *Lactobacillus* enriched in the PEP group was positively correlated with the secretions of cytokines (IL-1, IL-6, TNF-α), indicating a strong immunomodulatory effect. This is consistent with the previous report’s findings [53]. *Akkermansia* was positively correlated with the anti-inflammatory factor (IL-10). As shown in Figure 6C, four genera were positively correlated with propionic acid, isobutyric acid, butyric acid, valeric acid, isovaleric acid, and total SCFAs, including *Akkermansia* and *Lactobacillus*. *Akkermansia* and *Lactobacillus*, the core genera of the two SPAE groups and PEP-L group, have previously been reported to regulate the synthesis of SCFAs [44]. This is also supported by the results of this study, whereas the abundance of *Ruminococcus*, *Clostridium_XVIII*, *Anaerotruncus*, and *Peptococcus* enriched in the Con group was negatively correlated with SCFAs. The above studies suggested that the gut microbiota of mice was related to immune and metabolic indicators. Meanwhile, it can be speculated that the prebiotic effects of SPAE may be related to SCFAs production and immune modulation.

## 4. Conclusions

SPAE is a novel material that exhibits similar biological activity to PEP, and takes on a more uniform particle size distribution than the PEP. SPAE exhibited characteristic peaks at 843 cm^−1^, 1105 cm^−1^, and 1149 cm^−1^ in the FTIR spectra, indicating the presence of α-glycosidic bond and pyranose. Meanwhile, SPAE showed superior DPPH antioxidant activity with an EC_50_ value equaling 1.30 mg/mL at pH 3 and excellent digestion resistance after in vitro study. Analysis of the gut microbiota revealed an increase in SCFAs-producing *Akkermansia* by SPAE supplementation in mice. Conversely, the abundance of *Ruminococcus* and *Clostridioides* was reduced. Correlational analysis revealed positive associations between multiple microbiota and immune indexes as well as SCFA. *Akkermansia* enriched in the SPAE group was the key microbiota to positively promote the anti-inflammatory factor (IL-10) and SCFAs (propionic acid, isobutyric acid, butyric acid, valeric acid, and isovaleric acid). 

## Data Availability

Data is contained within the article or Supplementary Material.

## Supplementary Materials

The following supporting information can be downloaded at: https://www.mdpi.com/article/10.3390/nu16111555/s1, Table S1. Variation of the reducing sugar during the in vitro digestion of SPAE and PEP; Figure S1. Effects of SPAE and PEP on organ indexes. A: body weight; B: brain index; C: liver index; D: spleen index; E: thymus index; F: pancreas index. Different lowercase letters indicate significant differences in the different treatment groups at the level of *p* < 0.05.
