# Supplementary material for: Evaluation of Physicochemical Properties and Prebiotics Function of a Bioactive Pleurotus eryngii Aqueous Extract Powder Obtained by Spray Drying
Source: Nutrients. 2024 May 21;16(11):1555. doi: 10.3390/nu16111555 (PMC11173815; doi:10.3390/nu16111555)
Supplement: Supplementary file 1 [file nutrients-16-01555-s001.zip › nutrients-2993738-supplementary.pdf]

Supplementary materials

Evaluation of physicochemical properties and prebiotics function of a bioactive *Pleurotus eryngii* aqueous extract powder obtained by spray drying

[1] College of Life Sciences, Fujian Agriculture and Forestry University, Fuzhou 350002, China; cjqsw05@fafu.edu.cn (J. C.); 3210537056@fafu.edu.cn (M.Z.); chenliding@fafu.edu.cn (L.C.); 12305014041@fafu.edu.cn (Y.D.)

[2] Gutian Edible Fungi Research Institute, Fujian Agriculture and Forestry University, Ningde 352200, China

[3] Sanya Institute of China Agricultural University, Hainan, China; cfyang07@cau.edu.cn (C.Y.)

[*] Correspondence: lijiahuansw05@fafu.edu.cn (Jiahuan Li) and shujing2008@fafu.edu.cn (Shujing Sun); Tel.: +86-591-83789492; Fax: +86-591-83789352

Table S1 Variation of the reducing sugar during the *in vitro* digestion of SPAE and PEP

Samples	SPAE（mg/mL）	PEP（mg/mL）
Dynamic gastric digestion		
0 min	0.4165±0.0629e	0.5452±0.0196d*
10 min	0.7988±0.0654d	1.1335±0.0445c**
20 min	0.8949±0.0499cd	1.2056±0.0437c**
30 min	1.0207±0.1370c	1.3376±0.0889b*
40 min	1.2322±0.0694b	1.3605±0.0179b*
60 min	1.5803±0.1713a	1.8073±0.0679a
Dynamic small intestinal digestion		
60 min	0.4086±0.1022c	0.5048±0.1100b
90 min	0.6068±0.0622b	0.7504±0.0934a
120 min	0.6339±0.0987b	0.7950±0.0142a*
150 min	0.8458±0.0343a	0.8287±0.0525a
180 min	0.2562±0.0546d	0.2533±0.0047c

Note: Different lowercase letters indicate significant differences at the same digestion stage at the $p < 0.05$ level; *indicates significant difference ($p < 0.05$) between two polysaccharides at the same digestion point, **indicates significant difference ($p < 0.01$) between two polysaccharides at the same digestion point.

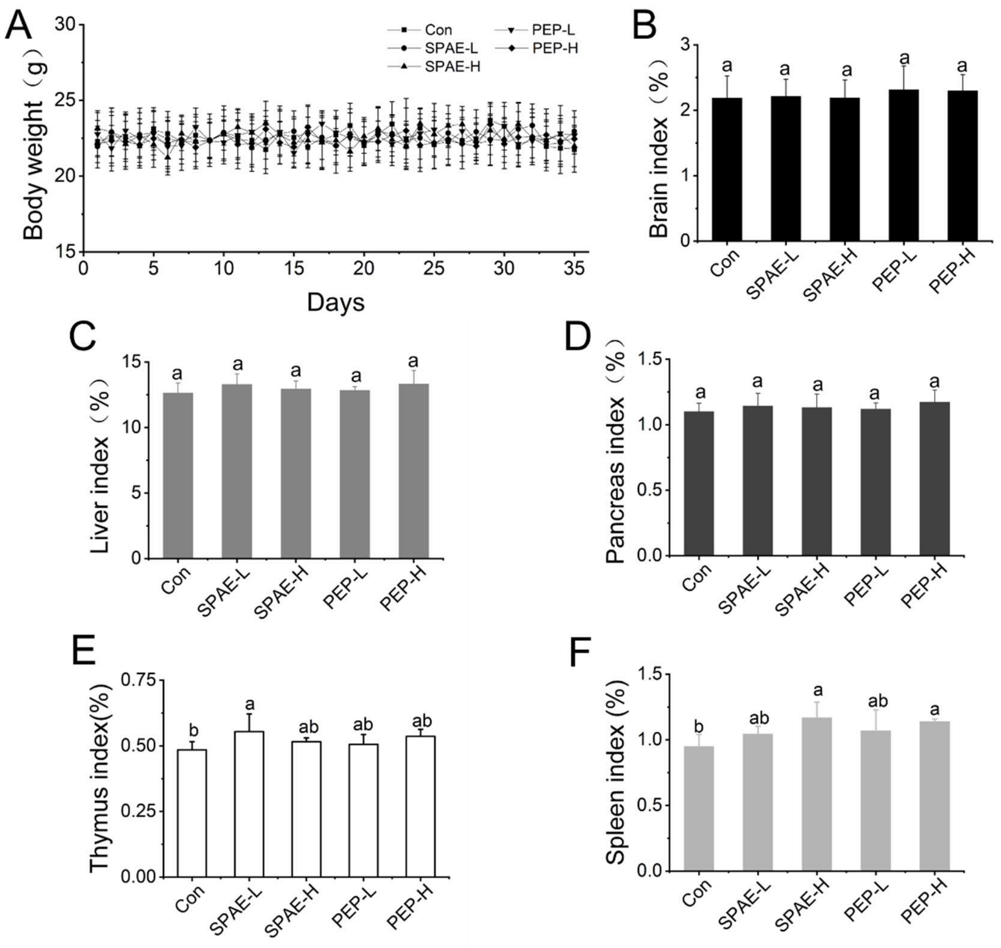

Figure S1 Effects of SPAE and PEP on organ indexes. A: body weight; B: brain index; C: liver index; D: spleen index; E: thymus index; F: pancreas index. Different lowercase letters indicate significant differences in the different treatment groups at the level of $p < 0.05$.
